# Exercise Improves Cardiac Dysfunction in D-Galactose-Treated Rats by Regulation of IGF-1-Humanin Pathway

**DOI:** 10.1155/jare/9949234

**Published:** 2025-09-27

**Authors:** Hesam Askarimoghadam, Farzaneh Rostamzadeh, Maryamossadat Mirtajaddini Goki, Elham Jafari, Mahboobeh Yeganeh-Hajahmadi

**Affiliations:** ^1^Cardiovascular Research Center, Institute of Basic and Clinical Physiology Sciences, Kerman University of Medical Sciences, Kerman, Iran; ^2^Physiology Research Center, Institute of Neuropharmacology, Kerman University of Medical Sciences, Kerman, Iran; ^3^Endocrinology and Metabolism Research Center, Kerman University of Medical Sciences, Kerman, Iran; ^4^Pathology and Stem Cell Research Center, Kerman University of Medical Sciences, Kerman, Iran

**Keywords:** D-galactose, heart, high-intensity interval training, humanin, IGF-1, moderate-intensity endurance training

## Abstract

**Introduction:** Humanin, a mitochondrial-derived peptide, decreases in the elderly. This study evaluated the effects of concurrent moderate-intensity endurance training (MIET) or high-intensity interval training (HIIT) with D-galactose injection on cardiac function, and the serum and heart levels of humanin and IGF-1 in Wistar male rats.

**Methods:** Left ventricular systolic pressure (LVSP), left ventricular end diastolic pressure (LVEDP), ±maxdp/dt, contractility index (CI) and, Tau were monitored by PowerLab system in CTL, CTL + MIET, CTL + HIIT, D-gal, D-gal + MIET, and D-gal + HIIT groups. The histopathological score, fibrosis, and humanin and IGF-1 levels were measured with hematoxylin & eosin, Masson's trichrome, and enzyme-linked immunosorbent assay, respectively.

**Results:** Histopathological score and heart fibrosis were reduced by HIIT and MIET in the D-gal group. LVSP, ±maxdp/dt, and CI were lower, while LVEDP and Tau were higher in the D-gal group than in the CTL group. MIET and HIIT alleviated the changes in LVSP, ±maxdp/dt, CI, LVEDP, and Tau. HIIT and MIET increased humanin levels in heart and serum of the D-gal group by modifying IGF-1 levels.

**Conclusion:** The study suggests HIIT and MIET may improve cardiac function by regulating the IGF-1-humanin signaling pathway.

## 1. Background

Longevity-related diseases are one of the most significant health concerns due to the increasing elderly population worldwide [1]. In addition to senescence and numerous disorders related to longevity, cardiovascular diseases (CVDs) also increase with age [2]. Moreover, CVDs are the main cause of mortality and morbidity in developed and developing countries [3]. A complex interaction of genetics and environmental factors leads to cellular dysregulation and cardiovascular disorders in aging [4]. Studies have shown that as people age, cardiovascular disorders such as stiffness of the arteries, left ventricular diastolic dysfunction, and reduction of cardiovascular capacity during exercise in healthy individuals occur [4, 5]. Cardiac aging is characterized by hypertrophy, fibrosis, damaged cell accumulation, and mitochondrial dysfunction [6, 7]. Endothelial dysfunction, which increases with aging, along with the formation of atherosclerotic plaques, reduces blood flow to the heart. The resulting ischemia may cause angina pectoris, acute myocardial infarction (MI), or even sudden death [6].

Aging-related diseases are associated with mitochondrial dysfunction, indicating that this organelle plays a vital role in maintaining cellular homeostasis [7]. Mitochondria play a key role in apoptosis, oxidative stress, and calcium homeostasis [8]. In addition, mitochondria produce specific peptides that regulate cell function via antioxidant, anti-inflammatory, and antiapoptotic properties [9].

Humanin, mitochondrial open reading frame of the twelve S-c (MOTS-c), and small humanin-like peptides (SHLPs) are mitochondrial-derived peptides (MDPs). Humanin was the first MDP discovered in 2001 [10]. The naming was based on its ability to restore “humanity” in Alzheimer's patients. It preserves neurons against amyloid-beta toxicity [11]. Humanin is a small peptide with 21 or 24 amino acids, depending on the place of translation, either in mitochondria or in the cytoplasm [8].

It has been shown that serum levels of humanin decrease with aging [11]. Induction of humanin expression or injection of humanin analog led to an increase in lifespan in worms [12] and improved health and metabolic indices in mice [13]. Subsequent studies have proven the beneficial properties of humanin in age-related disorders [14]. Humanin has protective impacts on mitochondrial function, helping to maintain cellular energy production and preventing oxidative stress [15]. Humanin reduces myocardial cell injuries induced by hydrogen peroxide and improves mitochondrial disorder by augmenting antioxidant defense systems [14] and inhibiting Complexes I and III of the electron transport chain activities [15]. Humanin reduces myocardial cell death and infarct area, preserving cardiac function after MI in an ischemia-reperfusion injury model by stimulating the expression of antioxidant enzymes [16].

The expression of humanin is regulated by various factors, including stress and insulin-like growth factor-1 (IGF-1) and growth hormone (GH). This relationship has been confirmed in mice and humans [13, 17]. On the other hand, GH and IGF-1 are important in regulating lifespan. In animal models, it has been shown that reducing the GH/IGF-1/insulin signaling pathways significantly increases lifespan [18]. However, in humans, the data are conflicting [19, 20]. Evidence shows that this pathway plays an essential role in the pathogenesis of age-related diseases such as cancer, dementia, CVD, and metabolic disease [21, 22]. Targeted interventions can improve health and quality of life in the elderly. Exercise is an effective nonpharmacological strategy that has preventive, therapeutic, and rehabilitative properties. Endurance and resistance exercises, or their combination, have positive effects on cardiovascular function [23]. Regular exercise is related to reducing the risk of CVDs [24]. Identification of molecular mechanisms has suggested that exercise can potentially reduce or reverse some aspects of the aging process in the heart [25]. Humanin is an exercise-sensitive mitochondrial peptide [25]. In young men, serum and muscle levels of humanin increase in response to acute high-intensity and short-term intermittent high-intensity exercise [25].

This study highlighted that moderate-intensity endurance training (MIET) and high-intensity interval training (HIIT) improve cardiac function in D-galactose-induced aging by regulating IGF-1 levels, which regulate the release of humanin, thereby reducing cardiac fibrosis and improving mitochondrial quality. The D-galactose injection model was selected because this model effectively recapitulates the multifaceted mechanisms underlying cardiac aging such as enhanced oxidative stress and inflammatory responses, disruption of cardiomyocyte autophagy and apoptosis, structural remodeling of cardiac tissue, and subsequent impairment of cardiac function [26].

## 2. Materials and Methods

### 2.1. Materials

The Ethics Committee of Kerman University of Medical Sciences approved the experimental protocol (ethics code IR.KMU.AEC.1401.025). Wistar male rats (220–250 g, 6–8 weeks) were obtained from Kerman University of Medical Sciences. The animals were kept under conventional conditions (12 h of light and 12 h of darkness) with free access to water and normal food. The following materials were also used in this study: Humanin enzyme-linked immunosorbent assay (ELISA) kit from CD Creative Diagnostics, USA (Cat. No.: DEIA10628); IGF1 ELISA kit from R&D Systems, USA (Cat. No: DY791).

**Figure 1:**
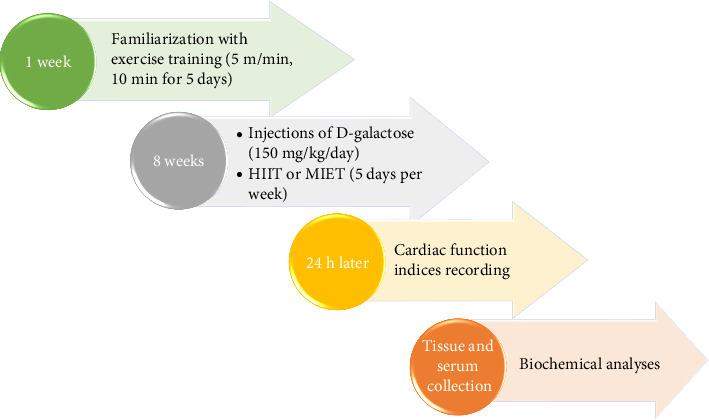
A flowchart depicting the experimental protocol.

The experimental groups (7 rats in each group) included the following: Control (CTL) group: Rats received subcutaneous injections of vehicle (saline) once daily for 8 weeks [27]. MIET group: Rats received subcutaneous injections of vehicle once daily for 8 weeks and underwent HIIT 5 days per week for 8 weeks. HIIT group: Rats received subcutaneous injections of vehicle once daily for 8 weeks and underwent HIIT 5 days per week for 8 weeks (MIET and HIIT programs are explained below). D-galactose (D-gal) group: Rats received subcutaneous injections of D-galactose (150 mg/kg/day) once daily for 8 weeks [28]. D-gal-MIET group: Rats received subcutaneous injections of D-galactose (150 mg/kg/day) and performed MIET concurrently for 8 weeks. D-gal-HIIT group: Rats received subcutaneous injections of D-galactose (150 mg/kg/day) and performed HIIT concurrently for 8 weeks (Figure 1)

### 2.2. Animal Exercise Program

To reduce stress and acclimate the animals to treadmill, rats first underwent low-intensity familiarization training (5 m/min, 10 min for 5 days). After that, to estimate the maximal running speed (*V*_max_), animals ran at an initial speed of 6 m/min with an increment of 2 m/min every 2 min until exhaustion [29]. The maximum running speed was used for calculating VO_2max_ [29]. HIIT was performed 5 days per week for 8 weeks according to the protocol in Table 1 [30]. The MIET program was conducted at 60%–65% of maximum VO2 for 8 weeks and 5 days per week. The duration of exercise gradually increased from 15 to 50 min, and the speed gradually increased from 10 to 35 m per minute (Table 2) [31].

### 2.3. Recording of Cardiac Function Parameters

Twenty-four hours after the last session of training, under anesthesia induced with sodium thiopental (50 mg/kg), cardiac function parameters were monitored. During data recording, dept of anesthesia were continuously monitored by toe-pinch, corneal reflex, heart rate, and respiratory rate. In cases where a decrease in anesthesia depth was observed, a supplemental dose of anesthetic was administered to maintain an adequate level of anesthesia.

Using a catheter, which was filled with heparinized saline (7 U/mL), inserted into the left ventricle (LV) via the right carotid artery, we measured cardiac function and heart contractility indices (CIs), including left ventricular systolic pressure (LVSP), left ventricular end-diastolic pressure (LVEDP), maximum rate of increase in left ventricular pressure during systole (+maxdp/dt), maximum rate of decrease in left ventricular pressure during diastole (−maxdp/dt), CI, and Tau (left ventricular diastolic time constant). The catheter was connected to the pressure transducer and then to a PowerLab system (8-channel; ADInstruments, Australia). After an establishment period of 10 min, the recording continued for 10 min. Tracheal cannulation was performed when needed to ensure adequate ventilation with animal ventilator [32].

### 2.4. Preparation of Heart Tissue and Serum

After recording cardiac function indices, blood was drawn from the heart under deep anesthesia and kept at room temperature for 20 min. Subsequently, clotted blood centrifuged at 2000–4000 rpm for 15 min and serum was separated and stored. The extracted hearts and lungs were rinsed with cold saline, and the LV plus the septum were gently separated and weighed. The ratio of heart, LV, and lung weight to body weight was calculated. The tissue samples were stored at −80°C for biochemical analyses.

### 2.5. Histopathological Changes and Fibrosis in Heart Tissue

To evaluate morphological changes and fibrosis, the extracted heart was fixed in 10% formaldehyde for 24 h. Subsequently, 5-μm slices were obtained from paraffin blocks of the LV and stained with hematoxylin-eosin (H&E) for assessment of myocyte damage, or with Masson's trichrome to identify fibrosis. The observations were graded with a total of 12 histological scores as follows: no changes, + mild (focal myocyte damage or small multifocal degeneration with slight inflammatory processes), ++ moderate (extensive myofibrillary degeneration and/or diffuse inflammatory processes), and +++ marked (necrosis with diffuse inflammatory processes) [32]. The fibrosis was quantified in 5 fields of each slice. The percentage of fibrosis was measured by ImageJ software [33]. A blinded pathologist assessed and reported the results using a light microscope (Olympus CX33, Japan).

### 2.6. Measuring the Heart and Serum Levels of Humanin and IGF-1

The levels of humanin and IGF-1 in serum and heart tissue were quantified using ELISA kits according to the manufacturer instructions. In brief, 20 mg of the LV tissue was homogenized on ice using lysis buffer containing protein inhibitor. The homogenate was centrifuged at 14,000 rpm for 15 min at 4°C. For measuring humanin, 50 μL and for measuring IGF-1, 100 μL of sample (serum or supernatant) containing the desired antibodies were loaded into the wells. Then, a washing step was performed to wash the other analytes. In the next step, a substrate was added, resulting in a blue color development proportional to the amount of humanin and IGF-1 in the sample. Finally, the reaction was stopped by adding the stop solution, and the amount of yellow color was assessed at a wavelength of 450 nm by the ELISA reader. Abundance of targeted protein was normalized to total protein in a sample (tissue sample). The levels of unknown proteins in samples were calculated using a standard curve.

### 2.7. Statistical Analysis

Continuous and numerical data in the tables and figures are presented as mean ± SEM. After checking normality with the Shapiro–Wilk test, the one-way ANOVA test was used for comparisons among groups, followed by the Tukey post hoc test. The categorical data (histopathological score) were analyzed by nonparametric tests. A linear regression was conducted to predict the association between humanin and IGF-1 changes. A *p* value < 0.05 was considered significant.

## 3. Results

### 3.1. The Effects of MIET and HIIT on Histopathological Changes and Fibrosis in the Heart

**Figure 2:**
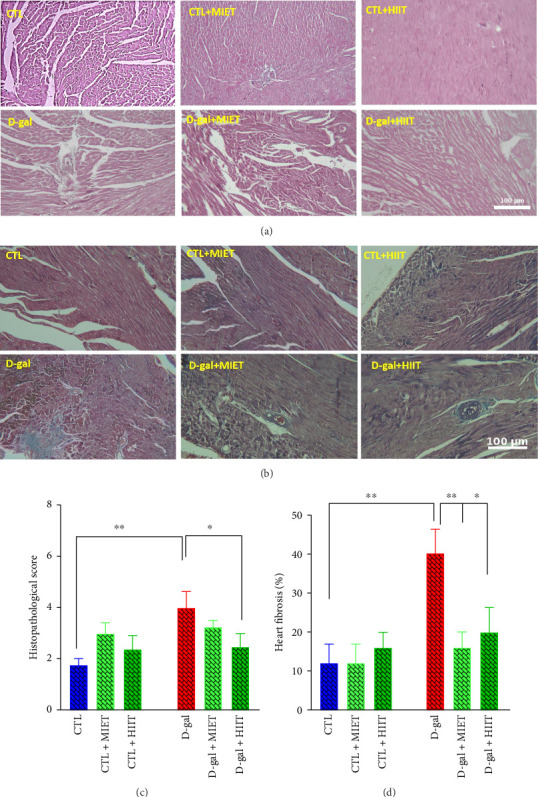
The effects of moderate-intensity endurance training (MIET) or high-intensity interval training (HIIT) on histopathological score and fibrosis in the heart of D-gal-treated rats. Histopathological score and fibrosis in one animal in each group (a, b). The quantification of heart histopathological score and fibrosis in each group (c, d). Magnification = x100. The values represent the mean ± SEM (*n* = 5). ^∗^*p* < 0.05 and ^∗∗^*p* < 0.01 vs. CTL and D-gal groups.

H&E and Masson's trichrome staining were performed to determine the preventive effects of HIIT and MIET on morphological damage and fibrosis in the heart caused by D-gal injection. The histopathological score and fibrosis were higher in the D-gal group than in the CTL group (*p* < 0.01). HIIT decreased the histopathological score (*p* < 0.05) in the D-gal group. Fibrosis was lower in the D-gal + HIIT (*p* < 0.05) and D-gal + MIET (*p* < 0.01) groups than that in the D-gal group (Figure 2).

Body weight was lower in the CTL + HIIT and D-gal + HIIT groups than in the CTL and D-gal groups (*p* < 0.05), respectively. Heart weight/body weight (HW/BW) and left ventricular weight/body weight (LVW/BW) were higher in the CTL + HIIT and CTL + MIET groups than those in the CTL group (*p* < 0.05). The lung weight/body weight ratio did not differ among groups (Table 3).

### 3.2. The Effects of MIET and HIIT on Cardiac Indices

**Figure 3:**
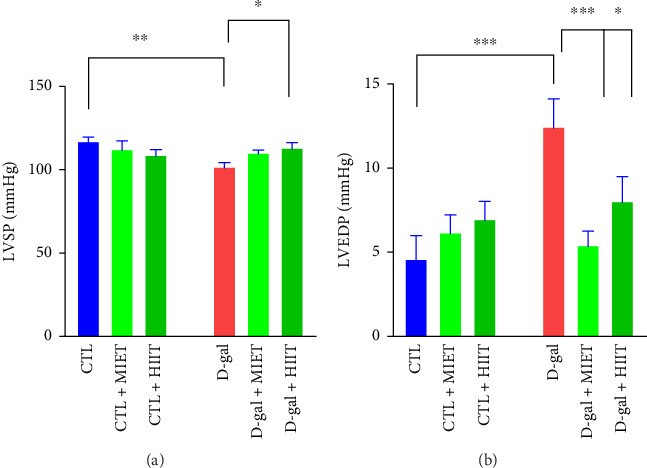
The effects of moderate-intensity endurance training (MIET) or high-intensity interval training (HIIT) on (a) left ventricular systolic pressure (LVSP) and (b) left ventricular end diastolic pressure (LVEDP) in D-gal-treated rats. The values represent the mean ± SEM (*n* = 7). ^∗^*p* < 0.05, ^∗∗^*p* < 0.01, and ^∗∗∗^*p* < 0.001 vs. CTL group and D-gal group.

LVSP (*p* < 0.01) was lower and LVEDP (*p* < 0.001) was higher in the D-gal group than those in the CTL group. MIET restored the reduction of LVSP in the D-gal group (*p* < 0.05). LVEDP was recovered by MIET and HIIT (*p* < 0.001, *p* < 0.05) (Figure 3).

**Figure 4:**
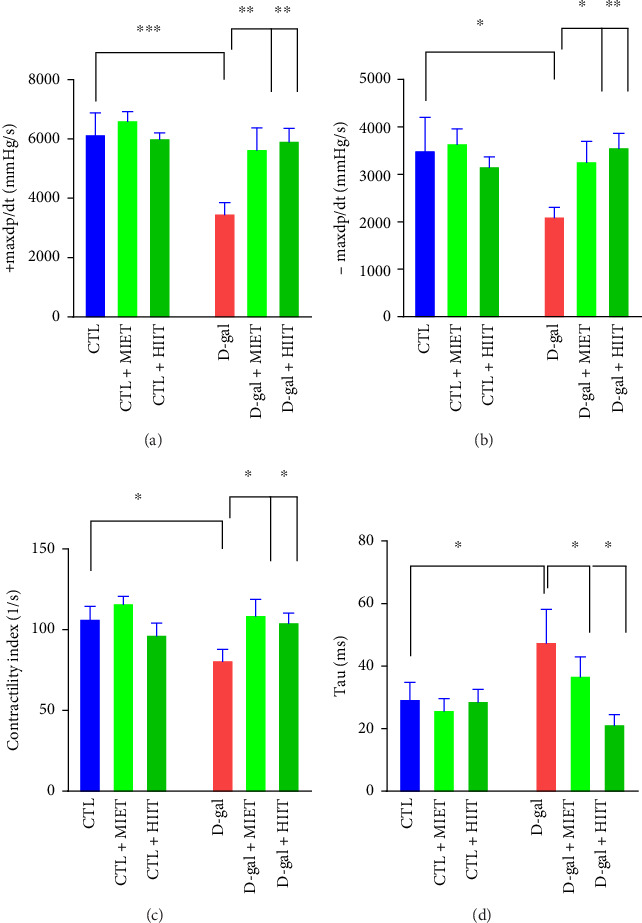
The effects of moderate-intensity endurance training (MIET) or high-intensity interval training (HIIT) on (a) +maxdp/dt, (b) −maxdp/dt, (c) contractility index (CI), and (d) tau in D-gal-treated rats. The values represent the mean ± SEM (*n* = 7). ∗*p* < 0.05, ∗∗*p* < 0.01, and ∗∗∗*p* < 0.001 vs. CTL group and D-gal group.

The +maxdp/dt and −maxdp/dt values were lower in the D-gal group than in the CTL group (*p* < 0.001 and *p* < 0.05). MIET and HIIT recovered the +maxdp/dt and −maxdp/dt values (*p* < 0.05 to *p* < 0.01). CI also decreased in the D-gal group compared to the CTL group (*p* < 0.05). MIET (*p* < 0.05) and HIIT (*p* < 0.01) diminished the reduction of CI values in D-gal-treated rats (*p* < 0.01). There was an increase in tau in rats that were treated with D-gal (*p* < 0.05). MIET and HIIT (*p* < 0.05) decreased tau in the D-gal group (Figure 4).

### 3.3. The Effects of MIET and HIIT on the Humanin and IGF-1 Levels in Serum and Heart

**Figure 5:**
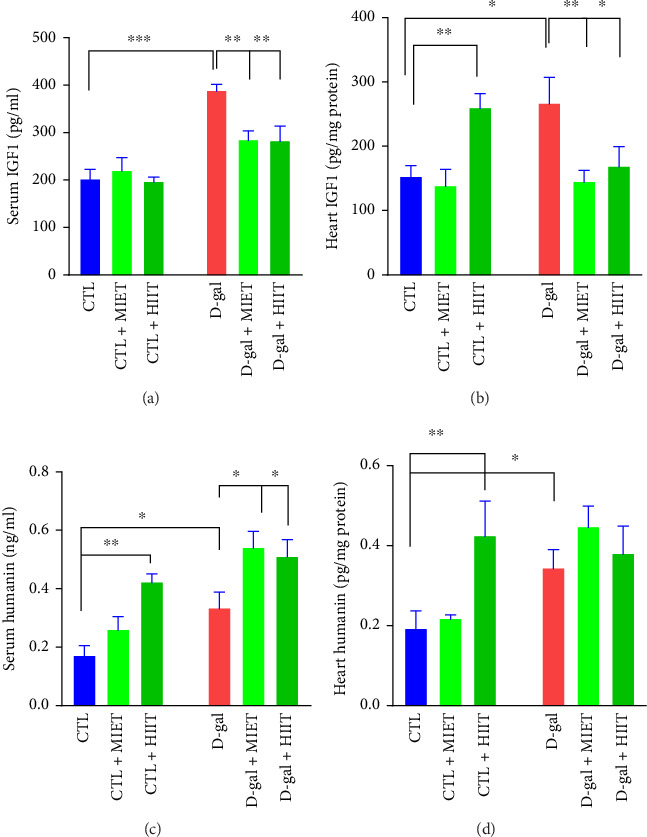
The effects of moderate-intensity endurance training (MIET) or high-intensity interval training (HIIT) on (a, b) heart and serum levels of humanin and (c, d) heart and serum levels of IGF-1 in D-gal-treated rats. The values represent the mean ± SEM (*n* = 5). ^∗^*p* < 0.05, ^∗∗^*p* < 0.01, and ^∗∗∗^*p* < 0.001 vs. CTL group and D-gal group.

**Figure 6:**
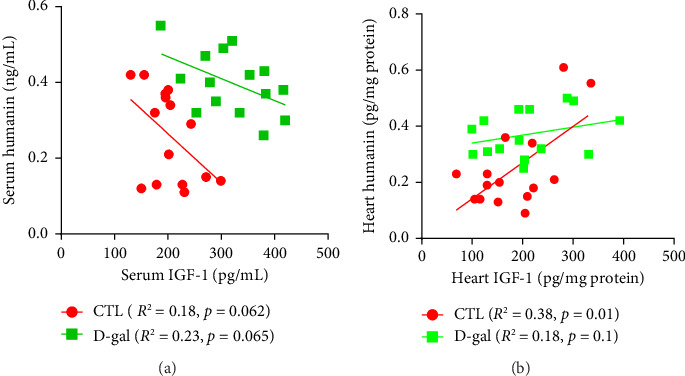
The linear regression-indicated relationship between humanin and IGF-1 in (a) serum and (b) heart in CTL and D-gal groups.

The ELISA results indicated the effects of MIET and HIIT on changes in serum and heart levels of humanin and IGF-1 in rats treated with D-gal. The serum (*p* < 0.001) and heart (*p* < 0.01) levels of IGF-1 was higher in the D-gal group than those in the CTL group. The serum level of IGF-1 decreased in response to MIET and HIIT (*p* < 0.01) in the D-gal-treated rats. In the heart, it significantly decreased in response to MIET (*p* < 0.01) and HIIT (*p* < 0.05) in the D-gal group (Figures 5(a) and 5(b)). The serum and heart levels of humanin increased in the D-gal group (*p* < 0.05, *p* < 0.01). MIET and HIIT increased humanin levels in the serum of rats treated with D-gal (*p* < 0.05) (Figures 5(c) and 5(d)). In the D-gal group, although both exercise intervention increased the levels of humanin in the heart, it was not significant. There was an increase in the levels of IGF-1 and humanin in normal rat that received HIIT (*p* < 0.01). Linear regression indicated a negative association between IGF-1 and humanin in serum of rats treated with D-gal (*R*^2^ = 0.23, *p*=0.065) and normal rats (*R*^2^ = 0.18, *p*=0.062). However, a positive association was observed between changes in humanin and IGF-1 in the heart of the CTL group (*R*^2^ = 0.38, *p*=0.01). In the D-gal group, this relationship (*R*^2^ = 0.18, *p*=0.1) was diminished (Figure 6).

## 4. Discussion

**Figure 7:**
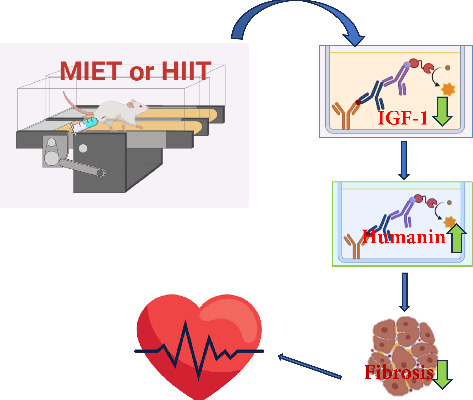
Moderate-intensity endurance training (MIET) or high-intensity interval training (HIIT) normalized increased IGF-1 levels, raised serum and heart humanin levels, and reduced heart damage and fibrosis in D-gal-treated rats, indicating cardioprotection linked to these molecular changes.

The findings of this study indicated both exercise interventions effectively, improved cardiac function indices including LVSP, left ventricular end-diastolic pressure, and contractility and relaxation indicators in the D-gal-treated groups. Exercise training also lessened the negative effects of D-gal on indicators of heart tissue damage and fibrosis. HIIT and MIET were able to ameliorate the increase in the heart and serum levels of IGF-1 observed in the D-gal group. Both types of exercise training also augmented the serum levels of humanin, and partly its heart levels, in rats treated with D-gal. The cardioprotection of exercise were probably associated with modified humanin and IGF-l levels in the serum and heart of D-gal-treated rats (Figure 7).

Consistent to our study, the positive effects of exercise on cardiovascular performance have been reported in the elderly [34–36]. Regular endurance training can significantly improve cardiovascular function in older men aged 60 to 70, similar to younger individuals [37]. These improvements are manifested by increased maximal oxygen intake, stroke volume, and cardiac output [37]. Exercise training effectively mitigates aging-associated cardiac apoptosis and fibrosis induced by D-gal treatment, improving overall cardiac structure [38]. It was shown that exercise plays a critical role in protecting and improving heart mitochondrial function during aging. Regular exercise preserves mitochondrial quality and reduces abnormal mitochondrial mitophagy seen in aging hearts [39–41].

Mitochondria, beyond their role in energy supply, produce peptides that regulate mitochondrial and cellular functions. The production of these peptides also dysregulates in age-related diseases [14]. It has been demonstrated that lower level of humanin, which is one of mitochondria-derived peptides, is associated with age and some age-related diseases in humans [14]. Moreover, humanin can be used as a biomarker for assessing mitochondrial function in CVD or as a therapeutic strategy in patients with endothelial dysfunction [8]. Contrast to the abovementioned studies, the results of the current study showed that the levels of humanin increased in the serum and heart of D-gal-treated rats. Although D-galactose induces cardiac aging by various mechanism including oxidative stress, inflammation, apoptosis, mitochondrial dysfunction, autophagy, and cardiac remodeling [26], this model may not completely simulate the aging process. The elevated levels of humanin in the D-gal group may be a compensatory response to oxidative stress that was created in the D-gal model rather than a direct aging [42]. According to our data, the improvement in the production and release of humanin in response to MIET and HIIT may contribute to the beneficial effects of exercise on heart function and structure. Studies have suggested that both acute and chronic exercise lead to increased humanin levels [43, 44]. Humanin plays a role in cellular protection, particularly in mitigating oxidative stress, inflammation, and apoptosis associated with physical activity [14, 45]. Recently, it has been shown that humanin has protective effects through the Notch signaling pathway in the heart [46].

Studies have shown that IGF-1 influences the expression and release of humanin [47]. Previous experiments have revealed that IGF-1 reduces humanin levels in rodents and humans [13]. Our data indicated that this negative association is obvious in serum in a healthy and aging model condition. However, in normal rats, IGF-1 and humanin changed in the same direction in the heart, and in D-galactose-treated rats, this relationship partly disturbed. It seems that the association between humanin and IGF-1 is complicated and may depend on the type of tissue, intervention, and age. Consistent to our study, it was reported that IGF-1 induces the humanin expression in Leydig cells of young rats, not older ones [47]. The increased humanin expression in response to IGF-1 in the heart of normal rats that received HIIT may be related to the protective effect of IGF-1 on mitochondrial quality [48]. Furthermore, besides IGF-1, other factors may regulate the expression and release of humanin including stress. Considering stress is an important regulating factor of humanin expression [49, 50], stress induced by exercise training may attribute to stimulation of humanin expression in the HIIT group. Exercise-induced stress on mitochondria [47, 48] may trigger an adaptive response, leading to increased humanin expression.

The current results indicated that both exercise modality partly modified IGF-1 and humanin concentration in a healthy and aged model. However, in contrast to D-gal-treated rats, in normal rats, only HIIT could change the heart IGF-1 and humanin levels. Therefore, the regulatory effects of exercise on IGF-1 and humanin modification may be influenced by age, tissue, and type and intensity of exercise training. Different types of exercise modalities have varying effects on IGF-1 [51–53]. It was shown that resistance training increased IGF-1 levels only when the participants were above 40 years [54]. Overall, evidence suggests that HIIT may offer greater health benefits on cardiovascular than traditional continuous aerobic exercise, as indicated by more substantial improvements in superoxide dismutase (SOD) activity, aerobic capacity, blood lipid profiles, and blood pressure [55].

In addition to the regulatory effect of IGF-1 on humanin gene expression, humanin and IGF-1 have some common effects, such as binding to insulin-like growth factor–binding Protein 3 (IGFBP-3) and activating common signaling pathways, which probably participate in cell survival mechanisms [47]. Considering positive effects of HIIT observed in this study on IGF-1 and humanin levels in the heart, it was suggested IGF-1 and humanin, potentially exerting synergistic beneficial effects on the healthy and aging heart. There are inconsistent findings about the changes of IGF-1 and effects of IGF-1 on the aging process. This study indicated the administration of D-galactose being associated with a rise in the level of IGF-1. In accordance with this study, it has been shown that inhibiting IGF-1/insulin signaling pathways or knockdown of IGF-1 increase lifespan in worm, rodents, and human [56]. In contrast to mice, enhancing IGF-1 signaling had negative effects on heart failure in rats [48]. However, other studies reported that the level of IGF-1 declines with age, and its decrease has been associated with age-related disorders such as CVDs and heart failure [57].

This study has several limitations. First, the observed associations between IGF-1, humanin, and cardiac function are correlative; we did not directly manipulate these molecules to establish causality. Second, the molecular mechanisms underlying these alterations remain unclear. We did not assess mitochondrial quality and function directly, which would have provided stronger support for the role of MDPs. Finally, although, the D-galactose has been used for aging investigation, it might not completely reveal the signaling pathways mediating the aging process.

## 5. Conclusion

This study investigated the preventive effects of two types of exercise intervention on humanin and IGF-1 levels in the heart and serum of rats that were treated with D-gal, providing novel insights into the cardioprotective mechanisms of exercise training. We revealed that D-gal-treated rats exhibited heart dysfunction. It was suggested that improving cardiac function in MIET and HIIT groups is associated with the modification of IGF-1 and humanin changes. The relationship between humanin and IGF-1 likely depends on the type of tissues and age. However, the specific mechanisms by which exercise influences humanin and IGF-1 expression and the exact association between humanin and IGF-1 are areas of ongoing research.

## Figures and Tables

**Table 1:** High-intensity interval training (HIIT) protocol.

Weeks	Frequency	Intervals	HIID (min)	LIID (min)	HIIV (*V*_max_)	LIIV (*V*_max_)	TETS (min)
1	5	4	2	1	80	50	12
2	5	4	2	1	80	50	12
3	5	6	2	1	85	50	18
4	5	6	2	1	85	50	18
5	5	8	2	1	90	50	24
6	5	8	2	1	90	50	24
7	5	10	2	1	95	50	30
8	5	10	2	1	95	50	30
7	5	10	2	1	95	50	30
8	5	10	2	1	95	50	30

Abbreviations: HIID, high-intensity interval duration; HIIV, high-intensity interval velocity; LIID, low-intensity interval duration; LIIV, low-intensity interval velocity; TETS, total exercise time in a session.

**Table 2:** Moderate-intensity endurance training (MIET) protocol.

Weeks	1	2	3	4	5	6	7	8
Speed *V*_max_	50–55%	50–55%	60–65%	60–65%	60–65%	60–65%	60–65%	60–65%
Time (min)	15	15	20	25	40	45	50	50

**Table 3:** The body weight and left ventricular and lung weight to body weight ratios in the study groups.

Groups variables	CTL	CTL + MIET	CTL + HIIT	D-gal	D-gal + MIET	D-gal + HIIT
BW (gr)	285 ± 12	255 ± 11	235 ± 12^∗^	274 ± 18	266 ± 12	242 ± 5.2^#^
HW/BW (mg/g)	2.8 ± 0.13	3.7 ± 0.28^∗^	3.6 ± 0.09^∗^	3.3 ± 0.19	3.2 ± 0.1	3.4 ± 0.05
LVW/BW (mg/g)	2.2 ± 0.14	2.54 ± 0.1^∗^	2.72 ± 0.12^∗^	2.38 ± 0.17	2.77 ± 0.13	2.65 ± 0.14
LW/BW (mg/g)	5.3 ± 0.28	6.1 ± 0.23	5.5 ± 0.17	5.4 ± 0.17	5.8 ± 0.30	5.9 ± 0.21

*Note:* LVW: left ventricle + septum weight; values are Mean ± SEM. *n* = 7 in each group.

Abbreviations: BW, body weight; LW, lung weight.

^∗^ *p* < 0.05 vs. CTL.

^#^ *p* < 0.05 vs. D-gal groups.

## Data Availability

Data are available on request from the authors.
